# Increased Fertilization Rates after In Vitro Culture of Frozen-Thawed Testicular Immotile Sperm in Nonobstructive Azoospermic Patients

**DOI:** 10.5402/2012/108576

**Published:** 2012-03-05

**Authors:** R. Nuñez-Calonge, S. Cortes, M. Gago, P. López, P. Caballero-Peregrin

**Affiliations:** Reproduction Unit, CLTNTCA Tambre, Calle Tambre no. 8, 28002 Madrid, Spain

## Abstract

*Objective*. To optimise the use of freeze/thaw testicular immotile spermatozoa from nonobstructive azoospermia patients and to analyse the outcome of intracytoplasmic sperm injection (ICSI) of such spermatozoa. *Methods*. Testicular specimens were retrieved and cryopreserved from forty patients with nonobstructive azoospermia and underwent one cycle with thawed spermatozoa (Group I) that led to pregnancy in sixteen cases. Twenty-four patients of group I underwent treatment with the same batch of thawed spermatozoa (Group II). For the first ICSI attempt, injection was performed when motile spermatozoa were found. In group II, injection was performed when maximum motility was reached. We compared mean of fertilization rate, embryo quality, clinical pregnancy rate and embryo implantation rate. *Results*. The mean percentage of motility was significantly higher in the group II than in the group I (18, 6 versus 8, 2). Group I showed a significant decrease in fertilization rates when compared with cryopreserved testicular spermatozoa in group II (54% versus 72%, *P* < 0.05). No difference was noted between the cleavage rate, embryo quality, clinical pregnancy rates and implantation rates among group II and I. *Conclusion*. Fecundation rate can be significantly improved after in-vitro culture and sperm selection of frozen-thawed immotile testicular spermatozoa in patients with nonobstructive azoospermia.

## 1. Introduction

It has been shown that the combination of human testicular sperm biopsy and intracytoplasmatic sperm injection (ICSI) is an efficient method for the treatment of male-factor infertility caused by azoospermia [1–4]. The successful use of testicular sperm extraction (TESE) procedures with ICSI led to the use of cryopreserved-thawed spermatozoa. The addition of sperm cryopreservation is valuable as it may reduce the number of procedures required for pregnancy success, and repeated testicular biopsies can then be avoided [5]. It seems that comparable success may be achieved with frozen-thawed and from testis spermatozoa [6–8]. It has been also reported that the motility of testicular spermatozoa can be improved after “in vitro” culture [7, 9, 10], although it is not known whether implantation and pregnancy rates are affected from such a practice [11]. Furthermore, it has also been observed [7] that the number of progressively motile spermatozoa was significantly increased after 72 h of culture, even if the total number of motile spermatozoa remained constant. Konc et al. [12] showed that condition of injected testicular spermatozoa has influence to embryo development, and even frozen/immotile testicular spermatozoa are able to induce/support fertilization and early embryo development. However, only limited data available connected with the efficacy of ICSI carried out with frozen nonmotile testicular spermatozoa. 

The aim of this study was to optimise the use of freeze/thaw testicular immotile spermatozoa from nonobstructive azoospermia (NOA) patients and to analyse the outcome of intracytoplasmic sperm injection (ICSI-TESE) of such spermatozoa. The outcome measures studied and compared were fertilization rate, embryo cleavage rate, clinical pregnancy rate and implantation rate.

## 2. Material and Methods

### 2.1. Patients

The data of sixty-four TESE-ICSI cycles performed in our centre in patients with NOA were evaluated prospectively. We studied forty couples in which the husband was diagnosed as having nonobstructive azoospermia (NOA) and only immotile testicular sperm cells were isolated by testicular sperm extraction (TESE). Testicular specimens were retrieved and cryopreserved and each couple underwent an ICSI cycle with thawed spermatozoa (Group I) that led to pregnancy in sixteen cases. Twenty-four patients of Group I subsequently underwent a consecutive cycle, which used frozen-thawed sperm that were retrieved in the original TESE procedure but were cryopreserved and stored until use (Group II). 

For the first ICSI attempt (Group I), injection was performed when motile spermatozoa were found. In most cases, viable sperm began to twitch after 2–4 hours in culture, and these cells were selected for ICSI. In all the twenty-four samples of Group II, one portion of the specimen was thawed the day before ICSI, and changes in postthaw motility after 2, 4, 6, 8, 12, and 24 h of culture at 37°C were recorded. In Group II, injection was performed when maximum motility was reached. 

### 2.2. Testicular Biopsy Preparation

The procedure of testicular sperm biopsy was similar to what has been already described [13] and the preparation of testicular spermatozoa was similar to the one reported by Liu et al. [14]. Briefly, the testicular tissue was placed in a Petri dish containing 2 mL sperm medium (Origio). After extraction of the connective tissue surrounding the seminiferous tubules with glass slides, an inverted microscope was used to detect the presence of spermatozoa at ×200 and ×400 magnifications (Diaphot, Nilkon). If a few spermatozoa were found, the contents of the Petri dish were transferred to a 15 mL Falcon tube. Prior to centrifugation, the larger debris of testicular tissue was removed from the tube, and the tube containing sperm suspension was centrifuged for 5 min at 300 g. After centrifugation, the supernatant was removed and the pellet was resuspended in about 100 *:* l IVF medium (Origio). A 15 *:* l aliquot of the sperm suspension was taken and put on a glass slide for examination of the initial motility of these spermatozoa. Motility of the spermatozoa was assessed and graded as nonmotile, weakly shaking, and progressively motile spermatozoa.

### 2.3. Ovarian Stimulation and Retrieval of Oocytes Used for ICSI

All female partners were stimulated using gonadotropin releasing hormone agonist or antagonist, human menopausal gonadotropin, and human FSH. Human chorionic gonadotropin was administered when optimal follicle development was achieved, as evaluated by serial transvaginal ultrasound and estrogen determinations. Oocyte retrieval was performed via a transvaginal approach with sonographic guidance 36 hours after human chorionic gonadotropin injection [7]. After oocyte retrieval, maturity of the oocytes was evaluated under the inverted microscope at ×400.

Cumulus-oocyte complexes were collected in IVF medium (COOK) and cultured for another 2 hours in the incubator before careful denudation using hyaluronidase (Origio). Immediately after this process, ICSI was started.

### 2.4. Embryo Assessment and Transfer

Fertilization was checked 16–18 h after ICSI. The fertilized oocytes were transferred and cultured in 50 mL droplets of Cleavage (COOK) medium covered by mineral oil (COOK), at 37°C in 6% CO_2_, 5% O_2_ and 89% N_2_ with maximal humidity in air. Cleavage was assessed after 48–72 h and embryo quality was evaluated prior to transfer. Embryo quality was evaluated on the basis of morphology and developmental stage. Embryos/oocytes were classified into five categories: (1) excellent, (2) good, (3) fair, (4) poor, and (5) degenerate. Embryo with normal morphology, which was being in a developmental stage appropriate for its age, was classified as transferable. Only category 1, 2, and 3 embryos were used for transfer and the maximum number of embryos transferred was three.

Embryo transfer was performed 48–72 h after oocyte retrieval using the Wallace catheter (The Edwards-Wallace Embryo Replacement Catheter, Sims, Portex Ltd., Kent, UK.).

Pregnancy was defined as a spontaneous rise in a *β*CG concentration at least 10 days after transfer. Clinical pregnancy implied the presence of an intrauterine gestational sac and fetal heart beat on an ultrasound performed at 7 weeks of gestation.

### 2.5. In Vitro Culture of Frozen-Thawed Testicular Spermatozoa

IVF medium (Origio) without additional supplements was used for culture of testicular spermatozoa. A 5 *:* l aliquot of testicular sperm suspension was added to each 10 *:* l droplet of IVF medium covered under mineral oil (COOK) and incubated at 37°C in an atmosphere of 6% CO_2_. In vitro culture of testicular spermatozoa was normally carried out without change of culture medium.

### 2.6. Motility of Fresh and Frozen-Thawed Spermatozoa

Washed spermatozoa were cultured in microdroplets under paraffin oil using bicarbonate-buffered medium containing 10% human serum in a 6% CO_2_ incubator at 37°C. 

Motility of frozen-thawed testicular spermatozoa was assessed under an inverted microscope (Nikon Diaphot) at ×400 magnification. One hundred testicular spermatozoa were counted in culture droplets for each sample. The percentage of nonmotile, weakly shaking, and progressively motile spermatozoa were recorded in both groups. Motility of frozen-thawed testicular spermatozoa in Group II was assessed at different intervals (1, 4, 6, 8, 12, and 24 h), and injection was performed when maximum motility was reached.

### 2.7. Cryopreservation and Thawing of Spermatozoa

After preparation of testicular spermatozoa, the same volume of cryoprotectant solution (TEST yolk buffer with glycerol, CRIOSPERM) was added to the testicular sperm suspension (volume/volume) and was gently mixed. This mixture was kept at 4°C for 45 minutes (in a refrigerator) and then the pellet of dry ice (Snowpack, MG Gas Products) was prepared. This is a modified cryopreservation method of Nagase and Niva [15], adapted to human spermatozoa [16, 17]. After the refrigeration, we prepared the mixture pills of 10 *:* l and placed then on dry ice for 2 minutes. Each pill was placed in 1.0 mL vials and plugged in liquid nitrogen. For thawing, the vials containing testicular spermatozoa were taken out from liquid nitrogen, and some of them were transferred into a 10 mL test tube and kept in a 37°C water bath for 3–5 minutes. About 5 mL medium (IVF, Origio) were added into the tube. After gentle mixing, the tube was centrifuged at 300 g for 5 min. After centrifugation, the supernatant was removed and the pellet was resuspended in about 50 *:* l IVF medium. The procedures of the in vitro culture of the frozen-thawed testicular spermatozoa and the examination of their motility were the same as the one described above for fresh testicular spermatozoa.

### 2.8. Statistic

A paired *t*-test was carried out to analyse the changes of motility before and after culture of fresh and frozen-thawed testicular spermatozoa. The chi-square test was used to compare the outcome of ICSI with fresh and frozen-thawed testicular spermatozoa. Probabilities <5% were considered significant.

## 3. Results

The average ages of the female and male patients were 35 + 2.9 (range 24–41) and 37.5 + 6.3 years (range 26–51). The average ages of the female and male patients were similar in the different treatment groups.

A total of 40 men underwent 40 TESE procedures.The result of the histology of testicular tissue indicated spermatogenic arrest in 80% of the cases. The spermatogenic arrest occurred at a different level of spermatogenesis (primary spermatocytes, 23%; spermatid, 77%). Sertoli-only syndrome was diagnosed in 20% of the patients. Male serum levels of FSH and testosterone were 23.4 + 12.1 mIU/mL and 11.7 + 7.6 nmol/L (mean + S.D.).

The main results of the treatment outcome are summarized in Table 1. No differences were found between the two groups in the days of gonadotrophin stimulation, number of ampoules/cycle, endometrial thickness, and estradiol level on the day of HCG administration. The number of oocytes retrieved did not differ among Groups I-II.

We found motile spermatozoa in both sperm groups. The mean percentage of motility was significantly higher in Group II (after culture, with the peaked) than in Group I (when spermatozoa reached some kind of motility) (18,6 ± 2,5 and 8,2 ± 3,2, resp.) (*P* < 0.01). In Group II, the motility improved markedly on the sixth hour of culture (Table 2).

Group I showed a significant decrease in fertilization rates when compared with cryopreserved testicular spermatozoa in Group II (54% versus 72%, *P* < 0.05, resp.). 

No differences were found in the embryo formation between oocytes injected with frozen-thawed testicular spermatozoa when reached some kind of motility (Group I) versus frozen-thawed testicular spermatozoa after culture, (94% versus 96%) (Table 3), neither quality of embryos between both groups (2,6 versus 2,7). 

Embryos generated from oocytes fertilized by ICSI using frozen-thawed testicular spermatozoa from Group I were transferred into 40 patients and 16 clinical pregnancy (16/40; 40%), and 14 deliveries (14/40; 35%) with 14 babies born were obtained. Embryos obtained from oocytes injected with frozen-thawed testicular spermatozoa from Group II were transferred into 24 patients. Out of them 10 became clinically pregnant (10/24; 41,6%) and 9 deliveries (9/24; 37,5%) with nine babies were obtained.

No difference was noted between the clinical pregnancy rates (40% versus 41,6%). There was no difference in implantation rates (25 versus 20,8) among Groups II and I (Table 3).

## 4. Discussion

Several authors have demonstrated that performing ICSI with fresh or frozen spermatozoa produces similar results [7, 8, 18, 19], and that freezing does not affect the spermatozoa [20]. 

Schlegel et al. observed in NOA patients, that in 30–38% of the cases the TESE procedure is unsuccessful. Thus, they and others underline the importance of the cryopreservation of the obtained testicular tissue in the clinical application of TESE.

Considering these results, repeated testicular biopsies are avoided in subsequent ICSI treatment cycles by cryopreservation of testicular spermatozoa in patients with NOA. Consequently, some groups have attempted to improve the protocols for testicular sperm freezing [5, 21, 22]. 

Our results support the observations of others that testicular tissue may be cryopreserved successfully without markedly reducing subsequent fertilization and implantation rates.

In our attempt to develop more efficient strategies, including freezing of testicular spermatozoa, we compared fertilization rates, cleavage rates, clinical pregnancy rates and implantation rates resulting from ICSI of frozen-thawed immotile testicular spermatozoa incubated 2–4 hours in a simple culture medium after thawing versus frozen-thawed immotile testicular spermatozoa incubated 2–24 hours in the same simple culture medium after thawing. 

Our results showed that after a few hours of culture of fresh and frozen-thawed testicular sperm samples obtained from patients with NOA, even without motile spermatozoa, the motility was significantly improved. The motility of cultured testicular spermatozoa reached a peak around 6 hours without the need for special media or treatment. The changes in motility patterns during in vitro culture of testicular spermatozoa has been observed previously [7], and practical implications of using in vitro matured testicular spermatozoa for ICSI were also described [10, 23]. The motility of cultured testicular spermatozoa in these cases reached a peak around 72 hours, and the results reveal that cryopreservation had an adverse effect on the survival rate of testicular spermatozoa, since the percentage of nonmotile spermatozoa significantly increased after cryopreservation [7]. These differences may be related to the procedures used in this study for in vitro culture and for cryopreservation of testicular spermatozoa. 

In order to improve the outcome of ICSI carried out with nonmotile spermatozoa, various methods have been described for the selection of the immotile but viable spermatozoa. These include the addition of pentoxifylline (PF), the laser touch technique, mechanical touch, and performing the hypo-osmotic swelling (HOS) test [24–27]. However, it has been argued, that using HOS test many live spermatozoa are to become nonviable after 30 min of incubation in the hypo- osmotic solution [27]. In addition, Casper et al. [27] reported lower fertilization rate after using HOS test protocol for ICSI. On the other hand, some studies showed that cAMP phosphodiesterase inhibitors are not beneficial in enhancing fertilization rates [28] or can have a negative effect on animal oocytes and embryos. They can cause meiotic arrest in mammalian oocytes [29], poorer development of mouse embryos [30, 31], 2-cell embryo block in some mouse strains [32, 33], or parthenogenetic activation of mouse oocytes [34]. On the basis of the findings that PF is not beneficial in enhancing fertilization rates and can be toxic for oocytes, it was suggested that PF should be used only in strictly selected patients [30, 31].

Conversely, it has never been described the influence of the effect of frozen-thawed human previously immotile testicular spermatozoa incubation time on intracytoplasmic sperm injection (ICSI) outcome.

In our study, frozen-thawed testicular nonmotile spermatozoa cultured for a period of 6–8 h resulted in a greater fertilization compared with testicular nonmotile spermatozoa without culture. No differences were found in embryo cleavage and clinical pregnancy outcomes between the two groups. 

The present findings are in agreement with other studies [35] that reported the motility of testicular spermatozoa was enhanced or initiated after in vitro culture and testicular biopsy culture may be an alternative to the use of motility stimulants to obtain motile spermatozoa for intracytoplasmic sperm injection. These results contrast with other reports that indicate that superior results are obtained with fresh TESE sperm rather than with TESE sperm cryopreservation [36, 37]. One possibility to account for these discrepancies is that the cryopreservation methods are variable between these groups and that freeze-thaw techniques for TESE sperm can significantly affect ICSI outcomes. Comparative studies to optimise tissue preparation, freezing protocols, and freezing media are rare and few attempts have been made to define the effects of cryopreservation on testicular spermatozoa. 

These data suggest that fecundation rate can be significantly improved after in vitro culture and sperm selection of frozen-thawed immotile testicular spermatozoa in patients with nonobstructive azoospermia. After all, a more rational management of testicular samples, in the case of frozen testicular spermatozoa, even though nonmotile spermatozoa, consists of improving sperm motility of samples by several hours of culture, after thawing and before ICSI. Higher fertilization rates with this method will produce more embryos. Therefore, it will be better embryo selection for transfer and embryo cryopreservation, offering the possibility for further attempts with one single sample.

We conclude that, although cryopreservation does impair motility, incubation of frozen/thawed testicular immotile sperm in cases of nonobstructive azoospermia appears to be beneficial.

## Figures and Tables

**Table 1:** General data.

	Group I	Group II	*P* value
No. of cases			
No. of cycles			
Female age			
Male age			
Days of gonadotrophin stimulation	10.5 ± 2.2	12.5 ± 3.2	NS
No. of ampoules/cycle	26.7 ± 10.1	31.7 ± 9.1	NS
Estradiol level on HCG day (pmol/L)	1395 ± 1571	1237 ± 1071	NS
Endometrial thickness (mm)	10.2 ± 2.4	9.8 ± 2.2	NS
Oocytes/cycle	11.2 ± 6.8	9.8 ± 7.6	NS
MII oocytes	377/495 (76.2%)	295/377 (78.2%)	NS

NS: not statistically different.

Values are mean ± SD. NS: not significant among all subgroups by the X^2^ double classification test, Fisher's exact test or the Students *t*-test where appropriated.

**Table 2:** Motility results with frozen-thawed spermatozoa on the moment of ICSI in both groups (Group I, when spermatozoa reached some kind of motility and Group II, after culture, with the peaked). Values are mean ± SD.

Time of in vitro culture	Percentage^a^ of spermatozoa that were motile	
	Group 1	Group 2
1	2 ± 2	3.3 ± 3
2	8.2 ± 2.5^a^	4 ± 5
4	—	11.8 ± 4
6	—	18.6 ± 3.6^a^
8	—	15 ± 18
12	—	10 ± 5
24	—	5 ± 10

^a^ *P* < 0.01.

**Table 3:** ICSI results with frozen-thawed spermatozoa on the moment of ICSI in both groups (Group I, when spermatozoa reached some kind of motility and Group II, after culture, with the peaked). Values are mean ± SD.

	Group I (*n* = 40)	Group II (*n* = 24)	*P*
Fertilization rate (%)	54	72	0.05
Range	42–75	68–92	
Cleavage rate (%)	94	96	NS
Range	60–100	70–100	
Grade 1,2 embryos	2,62	2,7	NS
Clinical pregnancy rate (%)	40	41,6	NS
	16/40	10/24	
Range	20,8	25	NS
Implantation rate (%)			
Deliveries	35	37,5	NS
	14/40	10/24	

## References

[1] Schoysman R, Vanderzwalmen P, Nijs M (1993). Pregnancy after fertilisation with human testicular spermatozoa. *Lancet*.

[2] Van Steirteghem A, Nagy Z, Liu J (1993). Intracytoplasmic single sperm injection with testicular sperm cell. *Annalen*.

[3] Devroey P, Liu J, Nagy Z (1994). Normal fertilization of human oocytes after testicular sperm extraction and intracytoplasmic sperm injection. *Fertility and Sterility*.

[4] Silber SJ, Van Steirteghem AC, Nagy Z, Liu J, Tournaye H, Devroey P (1995). High fertilization and pregnancy rate after intracytoplasmic sperm injection with spermatozoa obtained from testicle biopsy. *Human Reproduction*.

[5] Gianaroli L, Magli MC, Selman HA (1999). Diagnostic testicular biopsy and cryopreservation of testicular tissue as an alternative to repeated surgical openings in the treatment of azoospermic men. *Human Reproduction*.

[6] Prins GS, Dolgina R, Studney P, Kaplan B, Ross L, Niederberger C (1999). Quality of cryopreserved testicular sperm in patients with obstructive and nonobstructive azoospermia. *Journal of Urology*.

[7] Liu J, Tsai YL, Katz E, Compton G, Garcia JE, Baramki TA (1997). Outcome of in-vitro culture of fresh and frozen-thawed human testicular spermatozoa. *Human Reproduction*.

[8] Habermann H, Seo R, Cieslak J, Niederberger C, Prins GS, Ross L (2000). *In vitro* fertilization outcomes after intracytoplasmic sperm injection with fresh or frozen-thawed testicular spermatozoa. *Fertility and Sterility*.

[9] Balaban B, Urman B, Sertac A (1999). *In-vitro* culture of spermatozoa induces motility and increases implantation and pregnancy rates after testicular sperm extraction and intracytoplasmic sperm injection. *Human Reproduction*.

[10] Emiliani S, Van Bergh MD, Vannin AS, Biramane J, Verdoodt M, Englert Y (2000). Increased sperm motility after *in-vitro* culture of testicular biopsies from obstructive azoospermic patients results in better post-thaw recovery rate. *Human Reproduction*.

[11] Nijs M, Segal-Bertin B, Vandamme P (1997). In vitro culture of testicular spermatozoa: an improvement for the ICSI outcome?. *Journal of Assisted Reproduction and Genetics*.

[12] Konc J, Kanyó K, Cseh S (2008). The effect of condition/state of testicular spermatozoa injected to the outcome of TESE-ICSI-ET cycles. *European Journal of Obstetrics & Gynecology and Reproductive Biology*.

[13] Devroey P, Nagy P, Tournaye H, Liu J, Silber S, Van Steirteghem A (1996). Outcome of intracytoplasmic sperm injection with testicular spermatozoa in obstructive and non-obstructive azoospermia. *Human Reproduction*.

[14] Liu J, Garcia JE, Compton G Pregnancy after use of frozen-thawed human testicular spermatozoa by using intracytoplasmic sperm injection.

[15] Nagase H, Niwa T Deep freezing of bull semen in concentrated pellet form. I. Factors affecting survival of spermatozoa.

[16] Caballero P, Núñez R, Vázquez I, Dexeus JM, Barri PN (1993). Coordinación de un Banco de Semen. *Fertilidad, Análogos Gn RH-Periconceptología-Endoscopia*.

[17] Nunez-Calonge R, Cortes S, Gago M, García Segovia A, Peramo and Caballero P (2007). Optimización de los resultados de microinyección intracitoplasmática con espermatozoides congelados y descongelados procedentes de biopsia de testículo. *Revista Internacional de Andrología*.

[18] Gil-Salom M, Romero J, Mínguez Y (1996). Pregnancies after intracytoplasmic sperm injection with cryopreserved testicular spermatozoa. *Human Reproduction*.

[19] Küpker W, Schlegel PN, Al-Hasani S (2000). Use of frozen-thawed testicular sperm for intracytoplasmic sperm injection. *Fertility and Sterility*.

[20] Lin MH, Morshedi M, Srisombut C, Nassar A, Oehninger S (1998). Plasma membrane integrity of cryopreserved human sperm: an investigation of the results of the hypoosmotic swelling test, the water test, and eosin-Y staining. *Fertility and Sterility*.

[21] Allan JA, Cotman AS (1997). A new method for freezing testicular biopsy sperm: three pregnancies with sperm extracted from cryopreserved sections of seminiferous tubule. *Fertility and Sterility*.

[22] Crabbé E, Verheyen G, Tournaye H, Van Steirteghem A (1999). Freezing of testicular tissue as a minced suspension preserves sperm quality better than whole-biopsy freezing when glycerol is used as cryoprotectant. *International Journal of Andrology*.

[23] Zhu J, Meniru G, Craft I (1997). In vitro maduration of human testicular spermatozoa in patients with azoospermia. *Journal of Assisted Reproduction and Genetics*.

[24] Terriou P, Hans E, Giorgetti C (2000). Pentoxifylline initiates motility in spontaneously immotile epididymal and testicular spermatozoa and allows normal fertilization, pregnancy, and birth after intracytoplasmic sperm injection. *Journal of Assisted Reproduction and Genetics*.

[25] Aktan TM, Montag M, Duman S, Gorkemli H, Rink K, Yurdakul T (2004). Use of a laser to detect viable but immotile spermatozoa. *Andrologia*.

[26] de Oliviera NM, Sanchez V, Iesta SR (2004). Pregnancy with frozen-thawed and fresh tes- ticular biopsy after motile and immotile sperm microinjection, using the mechanical touch technique to assess viability. *Human Reproduction*.

[27] Casper RF, Meriano JS, Jarvi KA, Cowan L, Lucato ML (1996). The hypo-osmotic swelling test for selection of viable sperm for intracytoplasmic sperm injection in men with complete asthenozoospermia. *Fertility and Sterility*.

[28] Tournaye H, Van der Linden M, Van den Abbeel E, Devroey P, Van Steirteghem A (1993). Effects of pentoxifylline on in-vitro development of preimplantation mouse embryos. *Human Reproduction*.

[29] Downs SM, Eppig JJ (1986). The role of purines in the maintenance of meiotic arrest in mouse oocytes. *Tokai Journal of Experimental and Clinical Medicine*.

[30] Tournaye H, Van der Linden M, Van den Abbeel E, Devroey P, Van Steirteghem A (1993). Effect of pentoxifylline on implantation and post-implantation development of mouse embryos in vitro. *Human Reproduction*.

[31] Tournaye H, Van Steirteghem AC, Devroey P (1994). Pentoxifylline in idiopathic male-factor infertility: a review of its therapeutic efficacy after oral administration. *Human Reproduction*.

[32] Loutradis D, John D, Kiessling AA (1987). Hypoxanthine causes a 2-cell block in random-bred mouse embryos. *Biology of Reproduction*.

[33] Downs SM (1993). Purine control of mouse oocyte maturation: evidence that nonmetabolized hypoxanthine maintains meiotic arrest. *Molecular Reproduction and Development*.

[34] Scott L, Smith S (1995). Human sperm motility-enhancing agents have detrimental effects on mouse oocytes and embryos. *Fertility and Sterility*.

[35] Angelopoulos T, Adler A, Krey L, Licciardi F, Noyes N, McCullough A (1999). Enhancement or initiation of testicular sperm motility by in vitro culture of testicular tissue. *Fertility and Sterility*.

[36] Verheyen G, Nagy Z, Joris H, De Croo I, Tournaye H, Van Steirteghem A (1997). Quality of frozen-thawed testicular sperm and its preclinical use for intracytoplasmic sperm injection into in vitro-matured germinal-vesicle stage oocytes. *Fertility and Sterility*.

[37] Maier SH, Nesslauer T, Sterzik K, Hautmann RE (1999). Fresh sperms are superior for assisted fertilization (ICSI). *Journal of Urology*.

